# Lentiviral-Mediated Transgene Expression Can Potentiate Intestinal Mesenchymal-Epithelial Signaling

**DOI:** 10.1007/s12575-009-9014-z

**Published:** 2009-07-14

**Authors:** Adria D Dismuke, Aimee D Kohn, Randall T Moon, Melissa H Wong

**Affiliations:** 1 Department of Molecular and Medical Genetics, Oregon Health & Science University, Portland, OR, USA; 2 Cascade Cancer Center, Kirkland, WA, USA; 3 Howard Hughes Medical Institute, Division of Hematology, Department of Pharmacology, and the Center for Developmental Biology, Institute for Stem Cell and Regenerative Medicine, University of Washington School of Medicine, Seattle, WA, USA; 4 Departments of Dermatology; Cell and Developmental Biology; Knight Cancer Institute, Oregon Health & Science University, Portland, OR, USA

**Keywords:** lentivirus, mesenchymal-epithelial signaling, intestine, transgene expression, Wnt signaling, mouse

## Abstract

Mesenchymal-epithelial signaling is essential for the development of many organs and is often disrupted in disease. In this study, we demonstrate the use of lentiviral-mediated transgene delivery as an effective approach for ectopic transgene expression and an alternative to generation of transgenic animals. One benefit to this approach is that it can be used independently or in conjunction with established transgenic or knockout animals for studying modulation of mesenchymal-epithelial interactions. To display the power of this approach, we explored ectopic expression of a Wnt ligand in the mouse intestinal mesenchyme and demonstrate its functional influence on the adjacent epithelium. Our findings highlight the efficient use of lentiviral-mediated transgene expression for modulating mesenchymal-epithelial interactions in vivo.

## 1. Introduction

Epithelial-mesenchymal signaling networks are indispensible in vertebrates and are essential during early organogenesis and continuing throughout adulthood. Study of these complex pathways, including the Wnt, Notch, fibroblast growth factor, Bmp, TGF-β, Shh, and Notch signaling pathways, is complicated by the diverse interactions that exist between their respective ligands and receptors. However, understanding these signaling pathways is important, as many are disrupted in disease states or are required for maintaining tissue homeostasis by regulating stem cell maintenance, differentiation, migration, and polarity.

The mammalian intestine critically depends upon signaling between mesenchymal and epithelial cells. Many of the key developmental signaling pathways that function in an epithelial-mesenchymal capacity are implicated in maintaining the adult intestinal homeostasis. For example, the developmentally important Wnt signaling pathway is critical for proliferation and epithelial stem cell maintenance in the adult tissue [1]. In both the developing and adult intestine, a number of Wnt ligands are expressed in the mesenchyme and signal to receptors expressed in the intestinal epithelium [2]. While the importance of tightly regulated Wnt signaling in adult tissues is well-documented linking dysregulation of the pathway to hyperproliferation and cancer, our basic understanding of Wnt signaling on the ligand/receptor level is complicated by the large number of identified Wnt ligands and their receptors. In addition, ligands can bind to multiple receptor and/or co-receptor combinations to stimulate different downstream target gene expression. Therefore, due to this contextual influence, it has been difficult to dissect such complex interactions *in vivo*. Further, although many transgenic mouse lines with modifications in the Wnt signaling pathway exist, most harbor perturbations in downstream effectors, such as Apc or β-catenin. Moreover, the limited number of ligand knockout mouse lines in existence result in developmental defects that preclude analysis in the adult [3-5], and tissue-specific analyses are impeded by the lack of available knockout mice with conditional alleles. Mouse models exhibiting overexpression of Wnt ligands are also not readily available. While these transgenic and knockout mice could be generated, doing so for individual Wnt ligands and receptors in different tissues or cellular compartments would be inefficient and expensive. Although the Wnt signaling pathway exemplifies the intricate nature of mesenchymal-epithelial signaling, the challenges highlighted here also apply to studies in other signaling pathways. As such, it is important to explore alternative approaches for genetic manipulation to effectively study the role of mesenchymal-epithelial signaling pathways in homeostasis and disease.

Viral delivery of transgenes to either mediate ectopic gene expression or to dampen endogenous expression represents an attractive alternative approach as it can provide a rapid functional assessment of multiple receptors and ligands, individually or in tandem. Recent advances in the biosafety of lentiviral vectors make this approach a viable option, because non-replicative, self-inactivating viruses now cause little to no adverse effects on the infected subject [6-8]. Most importantly, this approach results in stable integration of the transgene into the mouse genome [7]. A track record for successful use of lentiviral-mediated gene delivery is apparent from studies in embryonic stem cells and somatic cells from a variety of organs, including the lung, pancreas, and stomach epithelium, as well as the liver [9,10]. Additionally, in utero viral injection of fetal rats results in a secondary transduction site in the intestinal epithelium [11]. Although this method provides an intriguing approach for epithelial transgene expression, *in utero* injection is a challenging technique that requires specialized equipment and skill. Further, this method is less attractive because generation of transgenic animals with intestinal specific promoters [12-15] is a standard approach that routinely accomplishes robust and uniform epithelial transgene expression and produces a transgenic line of mice. However, few systems exist for mesenchymal transgene expression, which is required to modulate paracrine signals originating in the mesenchyme. Our studies employ such an approach, and we have used the small intestine to demonstrate its effectiveness.

It is well-documented that the small intestine displays cell signaling across its defined epithelial and mesenchymal cellular compartments [2,12-14], offering an ideal system to display lentiviral-mediated transgene expression to modulate mesenchymal-epithelial crosstalk. In this study, we report effective lentiviral-mediated transgene delivery to mesenchymal cells and subsequent signaling to the epithelium. Functional levels of lentiviral-mediated WNT1 expression in the intestinal mesenchyme modulated transgene expression in the epithelium and resulted in a phenotypic readout.

## 2. Materials and Methods

### Mice

Mice were housed in a specific pathogen-free environment under strictly controlled light cycle conditions, fed a standard rodent Lab chow (PMI Nutrition International), and provided water ad libitum. FVB/N and Wnt-reporter TOPGAL mice [15] were purchased from The Jackson Laboratory (Stock #001800, #004623). Prior to lentiviral infection, mice were housed in a specific pathogen-free barrier room. After lentiviral infection, mice were moved to a standard barrier room. All procedures were performed in accordance to the Oregon Health and Science University Animal Care and Use Committee.

### Vector Production and in vivo Transduction

Lentiviruses were produced using the third generation HIV-pseudotyped system [6]. Transducing vectors contained DsRed (pSL35 = LMSCV-IRES-eGFP-DsRed) or human WNT1 (pSL35 = LMSCV-IRES-eGFP-WNT1), packaging vector pSL4, envelope vector pSL3, and rev-regulatory vector pSL5. pSL35 was modified to contain the murine stem cell virus promoter and the IRES-eGFP and multiple cloning site from pIRES2-eGFP (Clontech). High-titer lentiviruses were produced in 293T cells by FuGene 6-mediated transient co-transfection (Roche Applied Science) of each of the four vectors. Viruses were subsequently concentrated from conditioned media harvested 48 and 72 h after transfection by ultracentrifugation at 25,000 rpm in a Beckman SW28 swinging bucket rotor. Viral pellet was resuspended in sterile phosphate buffered saline (PBS) and titered by serial dilution and infection into HEK 293T/17 (ATCC #CRL-11268) cells, then expression titer determined by green fluorescent protein (GFP) expression by flow-cytometry analysis (FACSCalibur, Becton Dickinson). Viral pellet was resuspended in sterile PBS and titered by serial dilution and infection of 293T cells, followed by flow-cytometry analysis (FACSCalibur, Becton Dickinson) to determine the GFP expression titer. Viral titers were on average 5 × 10^7^ to 1 × 10^8^ infectious units/ml. All viruses were tested for replication ability by P24 assay and found to be negative (HIV-1 P24 enzyme-linked-immunosorbent serologic assay, PerkinElmer). For lentivirus transduction, postnatal day 1 TOPGAL and FVB/N mice were administered 5 × 10^6^ viral particles in a total of 50 μl, by intraperitoneal injection using a 30-gauge needle and analyzed 7, 14, 21, or 28 days later (Appendix).

### Analyses of Transgene Expression

The stomach, small intestine, colon, liver, kidney, brain, lung, spleen, heart, and skin were harvested and processed for mRNA expression analyses by quantitative reverse transcriptase-polymerase chain reaction (qRT-PCR) and for protein expression by immunoblot and immunohistochemistry as described below. For a more focused analysis of the intestine, epithelial and mesenchymal cellular compartments were independently isolated. For collection of epithelial fractions, the villus and crypt epithelium was differentially isolated using a modified Weiser preparation [17,18]. The remaining tissue was mechanically dissociated over a 10-mesh sieve (Bellco Tissue Sieve^®^, Bellco) to isolate the mesenchymal cellular compartment from the muscularis. The three individual cellular compartments (villus epithelium, crypt epithelium, and mesenchyme) were then snap-frozen in liquid nitrogen for future expression analyses.

### qRT-PCR and RT-PCR Analyses

RNA was isolated (QIAGEN RNeasy) and cDNA generated using Moloney murine leukemia virus-reverse transcriptase (Invitrogen) as previously described [19]. RT-PCR was performed using standard methods and primers specific to the human WNT1 gene (F-CTGCTCAGAAGGTTCCATCG, R-GCCTCGTTGTTGTGAAGGTT), GFP (F-CGGCATCAAGGTGAACTTC, R-TCACGAACTCCAGCAGGAC), and mouse β-actin (F-GAAGTACCCCATTGAACATGGC, R-GACACCGTCCCCAGAATCC). qRT-PCR analyses were performed using SYBR^®^-Green technology on an ABI 7900HT System (Applied Biosystems). All reactions were performed in triplicate in a reaction volume of 15 μl, using 100 ng of template DNA, 7.5 μl of a 2X SYBR^®^-Green Master Mix, 0.15U UDP-N-glycosidase (Invitrogen), and 900 nM forward and reverse primers. Data analysis using the ΔΔC_T_ (cycle threshold) approach was performed using the ABI Prism^®^ SDS 2.1 software. Cycle threshold values were determined for each gene, and each sample was normalized to the internal control gene, glyceraldehyde 3-phosphate dehydrogenase (Gapdh). Primers used are listed as follows: Gapdh (F-AAATATGACAACTCACTCAAGATTGTCA, R-CCCTTCCACAATGCCAAAGT), GFP (F-TCCAGGAGCGCACCATCTT, R-CGATGCCCTTCAGCTCGAT), DsRed (F-GAGATCCACAAGGCCCTGAAG, R-GTCCAGCTTGGAGTCCACGTA), LacZ (F-GATCTTCCTGAGGCCGATACTG, R-GGCGGATTGACCGTAATGG), c-Myc (F-AGCTTCGAAACTCTGGTGCATAA, R-GGCTTTGGCATGCATTTTAATT), EphrinB1 (F-AGGTTGGGCAAGATCCAAATG, R-AGGAGCCTGTGTGGCTGTCT), EphB2 (F-ACCTCAGTTCGCCTCTGTGAA, R-GGACCACGACAGGGTGATG), and EphB3 (F-TCTGACACTCAGCTCCAACGA, R-CCAGGCATCCAAAAGTCCA).

### Immunoblot Analyses

Whole intestinal tissue was harvested, flushed with cold PBS, snap-frozen in liquid nitrogen, lyophilized overnight, and subsequently ground into a powder with a mortar and pestle. Samples were resuspended in 1× sample buffer as described [20], then subjected to electrophoresis on a 4% stack/15% resolving acrylamide gel and transferred overnight at 20 V, 4 C to polyvinylidene fluoride membrane. For protein detection, the membrane was incubated with Odyssey^®^ Blocking Buffer (LI-COR Biosciences) for 1 h at 25 C, then probed with antibodies to GFP (1:1,000; Molecular Probes), DsRed (1:1,000; Clontech), Wnt1 (1:1,000, R&D Systems), or β-Actin (1:1,000, Santa Cruz). The Wnt1 antibody was raised against mouse, cross-reacts with the human protein, and has less than 5% cross-reactivity with Wnt3a and Wnt5a. Direct near-infrared detection was performed using appropriate species-specific secondary antibodies (rabbit IRDye 800, Rockland; mouse AlexaFluor 680, Molecular Probes), prior to being visualized on the Odyssey Infrared Imaging System (LI-COR Biosciences).

### Immunohistochemical Analyses

Liver, spleen, lung, and the intestine were processed for frozen sectioning and antibody staining as previously described [21]. GFP-expressing cells were identified in 5-μm-thick sections with antibodies to GFP (1:250; Molecular Probes) and fluorescent secondary antibodies (1:500, Cy5; Jackson ImmunoResearch); or were double-labeled with antibodies to DsRed (1:200; Clontech), Ki67 (1:200, AbCam), or β-galactosidase (β-gal; 1:500; Immunology Consultants Laboratory, Inc.); and visualized with Cy5- and FITC-conjugated secondary antibodies (Jackson ImmunoResearch). Slides were incubated with Hoechst 33258 (0.1 μg/ml; Sigma) nuclear stain. Images were captured on a Leica DMR microscope with a DC500 digital camera and IM50 Image Manager Software (Leica Microsystems). Images were overlaid using Adobe Photoshop CS2 or ACD Systems Canvas.

The number of crypt-villus units containing Wnt-receiving cells was determined in WNT1-injected and DsRed-injected mice by counting positive crypt-villus units on 6 × 5 μm tissue sections 125 μm apart (*n* = 3 each group) and represented as a ratio relative to total crypt-villus units. Statistical significance between experimental populations was determined using a Student's two-tailed, paired *t*-test. A *p* value <0.05 was considered statistically significant. Statistical analysis was performed using GraphPad Prism for Windows (GraphPad Software).

## 3. Results and Discussion

### Lentiviral-Mediated Transgene Expression in the Mouse

**Figure 1:**
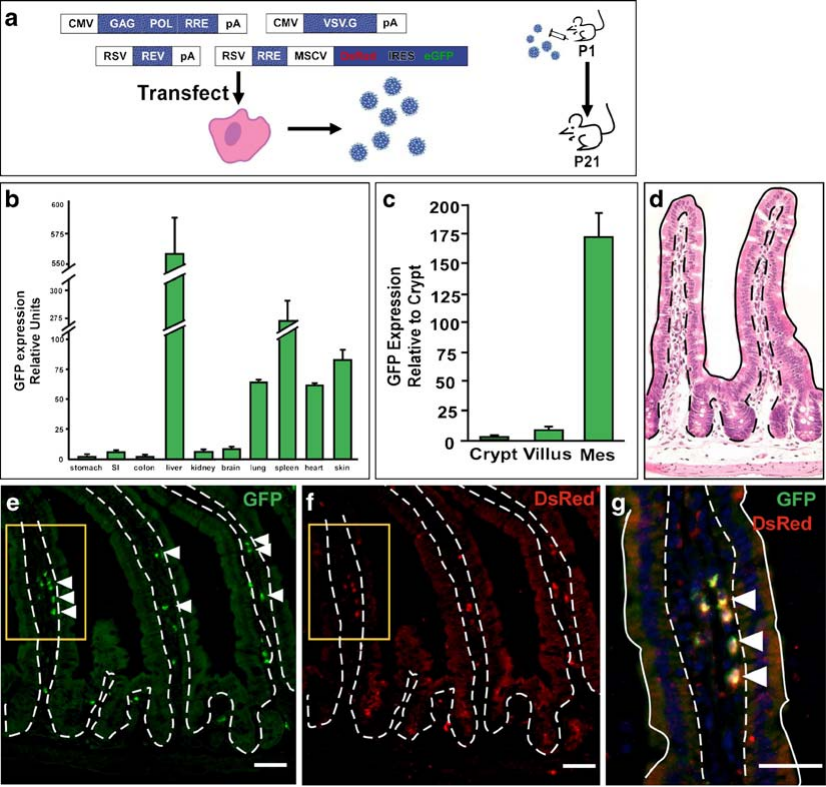
**Lentiviral-mediated gene delivery to the mouse small intestine**. **a** Schematic of virus production in 293T cells and mouse injection. Mice were injected at postnatal day 1 and sacrificed for analysis at later time points. **b** Green fluorescent protein (GFP) gene expression by quantitative reverse transcriptase-polymerase chain reaction (qRT-PCR) in the gastrointestinal tract and other organs. GFP expression is normalized to glyceraldehyde phosphate dehydrogenase expression and presented relative to levels in the stomach. Graph displays triplicate samples from representative animals. Data displayed as mean ± std. **c** GFP expression in isolated intestinal crypt and villus epithelium and mesenchymal cells by qRT-PCR. Graph displays triplicate samples from representative animal. Data displayed as mean ± std. **d** Hematoxylin and eosin stained normal intestinal crypt-villus unit. *Solid line* indicates apical epithelial border; *dashed line* indicates epithelial-mesenchymal border. **e–g** Co-labeling of DsRed-injected intestine with antibodies to GFP (*green*) and DsRed (*red*). *Yellow box* in e and f is enlarged in g. *Arrowheads* indicate dual expressing mesenchymal cells. *Dashed line* indicates epithelial-mesenchymal border. *Solid white line* in g marks the apical border. Bar = 25 μm.

To demonstrate that lentiviral-mediated delivery of transgenes represents an effective approach for modulating signaling pathways dependent upon mesenchymal-epithelial interactions, we characterized *in vivo* infection and mouse tissue expression patterns of a lentivirus harboring a bicistronic message encoding DsRed sequences upstream of an internal ribosomal entry site and enhanced GFP (DsRed-IRES-GFP). The virus was generated by co-transfecting three lentiviral packaging plasmids [6] with the DsRed-IRES-GFP transducing vector (Figure 1a). Lentiviral particles (5 × 10^7^) were delivered by intraperitoneal injection into wild-type mice at postnatal day 1 that were subsequently sacrificed and analyzed at postnatal day 21 (Figure 1a). Multiple tissues were isolated and analyzed by qRT-PCR for GFP expression (Figure 1b). Expression was highest in the liver and spleen, presumably due to the high percentage of blood-derived cells in these organs, but also detectable in the kidney, lung, skin, and the small intestine, as it has been demonstrated that intraperitoneal injection of substances effectively traffics to the blood compartment [22,23]. Mock-injected controls displayed no GFP expression since GFP is not endogenously expressed in wild-type mice. As we previously noted, the small intestine has well-defined epithelial and mesenchymal cellular compartments and known pathways exhibiting signaling crosstalk between the two distinct regions, offering an ideal system in which to continue our studies.

### Intestinal Transgene Expression is Restricted to the Mesenchyme

To identify the transgene-expressing cell population, we stained frozen intestinal sections from lentiviral-infected mice with antibodies to GFP and DsRed (Figure 1e–g). Co-localized expression of GFP and DsRed was restricted to the mesenchymal cells in both the crypt and villus. To confirm the mesenchyme-restricted expression pattern, cells from the mesenchyme and epithelial compartments were independently isolated by both dissociation of epithelial cells in ethylenediaminetetraacetic acid and mechanical dispersion of mesenchymal cells [17,18]. Restriction of lentiviral gene expression to the mesenchymal population was confirmed by qRT-PCR using primers specific to GFP and normalized to the housekeeping gene, Gapdh (Figure 1c). This compartment-specific expression allows for manipulation of paracrine signaling from the mesenchyme to the intestinal epithelium.

### Expression of WNT1 Ligand in the Mouse Intestinal Mesenchyme

**Figure 2:**
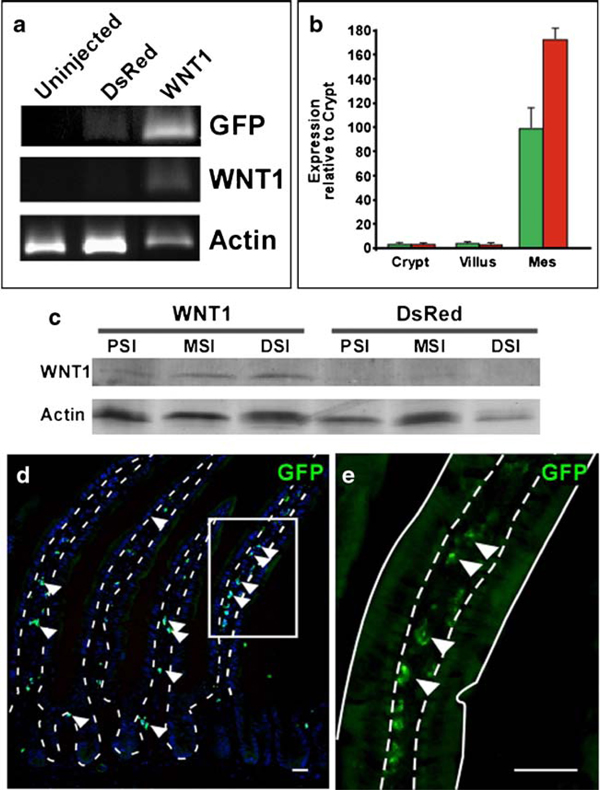
**Ectopic expression of WNT1 in the mouse intestine**. **a** Reverse transcriptase-polymerase chain reaction (RT-PCR) for intestinal expression of human WNT1, green fluorescent protein (GFP), and Actin in uninjected, DsRed-injected, and WNT1-injected mice. **b** Gene expression levels of GFP (*green*) and WNT1 (*red*) in isolated crypt and villus epithelium and mesenchymal cells by qRT-PCR. Levels normalized to internal reference gene and relative to crypt values. Samples from representative animal assayed in triplicate, data reported as mean ± std. **c** Immunoblot probed with antibodies to WNT1 and Actin from proximal, mid, and distal small intestine (*PSI*, *MSI*, and *DSI*) of WNT1- and DsRed-infected animals. **d** Tissue section from WNT1-injected mice stained with antibodies to GFP (*green*) and counterstained with Hoechst dye. *Arrowheads* indicate GFP-expressing mesenchymal cells. *White box* is enlarged in **e**. *Dashed line* represents epithelial-mesenchymal boundary; *solid line* represents apical epithelial border. Bar = 25 μm.

To show functional expression of a signaling molecule expressed by lentiviral infection, we chose to ectopically express the human WNT1 ligand in the intestinal mesenchyme. Although canonical Wnt ligands are present in the mouse intestine, Wnt1 is not endogenously expressed (data not shown; [2]). Stimulation of the Wnt/β-catenin signaling pathway has a well-characterized effect on the epithelium, resulting in hyperproliferation [24,25]. A WNT1-IRES-GFP lentivirus was generated and injected into mice as we described above. Similar to the DsRed lentivirus, WNT1 and GFP were expressed down the length of the intestine and were primarily restricted to the mesenchyme (Figure 2a, b). Immunoblot analysis for WNT1 revealed an expected 41 kD band in the proximal (PSI), mid (MSI), and distal small intestine (DSI) of infected mice (Figure 2c). Although the antibody was raised against a mouse peptide, it also recognizes the human protein. This is apparent with the lack of endogenous Wnt1 detection in the DsRed-infected compared to detectible levels in the WNT1-infected intestines, as this ligand is not expressed in the mouse intestine [2]. Further, GFP expression was detected by immunofluorescence in the mesenchyme, both near the crypts and on the villi (Figure 2d, e). Thus, the expression pattern was ideal to carry out studies using overexpression of ligands of mesenchymal origin.

### Functional Expression of WNT1 in the Mouse Intestine

**Figure 3:**
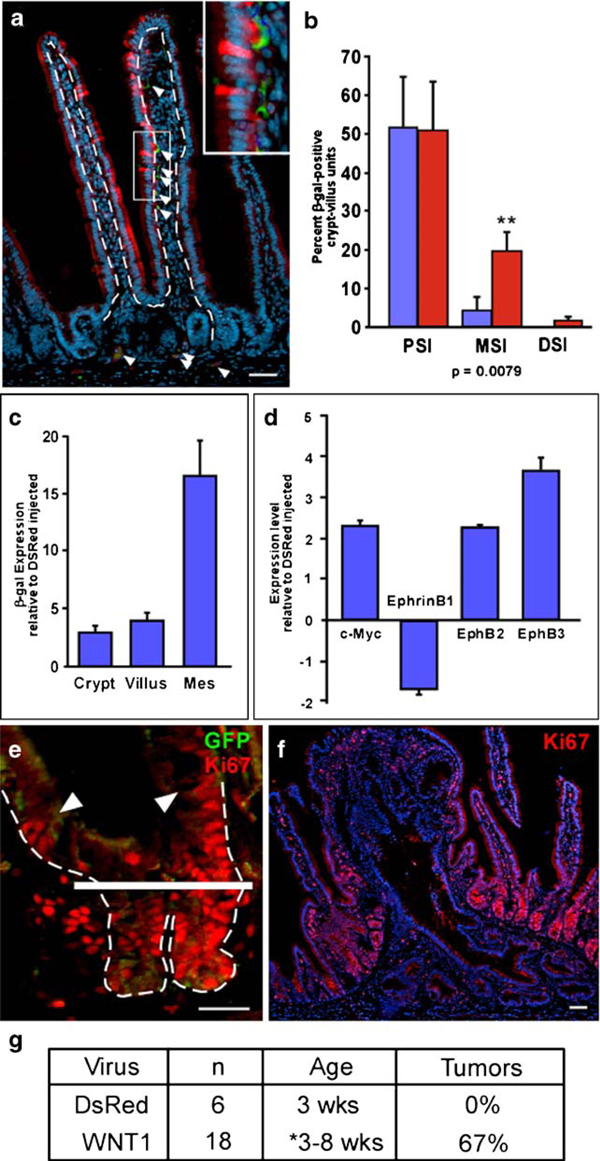
**Functional phenotype in WNT1-injected mice**. **a** Intestinal section stained with antibodies to β-galactosidase (β-gal; *red*) and green fluorescent protein (GFP; *green*), and counterstained with Hoechst dye (*blue*) in a WNT1-injected Wnt-reporter mouse. *Arrowheads* denote GFP-expressing WNT1-infected cells. Higher magnification of *white box in inset*. *Dashed line* represents epithelial-mesenchymal boundary. **b** Quantitation of intestinal Wnt-receiving cells in the proximal, mid, and distal small intestine (*PSI*, *MSI*, and *DSI*) by immunostaining with antibodies to β-gal. The number of crypt-villus units containing Wnt-receiving cells was counted in DsRed-injected (*blue bars*) and WNT1-injected (*red bars*) Wnt-reporter mice and represented as a ratio to total crypt-villus units counted (*n* = 3 for all three regions; *MSI*, *p* = 0.0079). **c** Quantitative reverse transcriptase-polymerase chain reaction (qRT-PCR) assay for LacZ expression in isolated intestinal crypt, villus, and mesenchymal cells from WNT1- and DsRed-infected Wnt-reporter mice. Graph displays triplicate samples from representative animals. Data displayed as mean ± std. **d** qRT-PCR for gene expression of canonical Wnt target genes: c-Myc, EphrinB1, EphB2, and EphB3. All qRT-PCR samples normalized to internal reference gene, represented relative to DsRed-infected cells and run in triplicate. **e** Intestinal crypt section from WNT1-injected mouse stained with antibodies to Ki67 (*red*) and GFP (*green*). *Solid white bar* indicates crypt-villus junction. *Arrowheads* denote Ki67-positive epithelial cells outside of normal proliferative zone. *Dashed line* indicates epithelial-mesenchymal boundary. **f** Polyp from *PSI* of WNT1-infected mouse stained with antibodies to Ki67 (*red*) and Hoechst (*blue*). Bar = 25 μm. **g** Quantitation of intestinal tumors from DsRed- and WNT1-injected mice.

Wnt-reporter mice (TOPGAL), a transgenic mouse line that expresses β-gal in response to a canonical Wnt signal [15], were injected with WNT1-IRES-GFP lentivirus to determine if lentiviral-mediated gene delivery could result in an increase in Wnt activation within the intestinal epithelial compartment. β-gal-expressing epithelial cells were detected in juxtaposition to GFP-expressing mesenchymal cells (Figure 3a). Additionally, an appreciable increase in the percentage of β-gal-positive crypt-villus units was detected in the MSI and DSI relative to control injected mouse intestines [17]; Figure 3b). Analysis by qRT-PCR confirmed the increase in β-gal reporter expression in WNT1 infected intestines, demonstrating a greater than fourfold increase in the epithelial compartment of both the crypt and villus (Figure 3c). Thus, lentiviral-derived ectopic expression of ligands can modulate transgene expression in the epithelium.

### Epithelial Response to Expression of WNT1

We further validated the canonical Wnt stimulation by examining known downstream target genes. As expected, the increase in Wnt-reporter expression was accompanied by an increase in Wnt target genes c-Myc, EphB2, and EphB3, and decreased EphrinB1 (Figure 3d; [12,13]). These results indicate not only that lentiviral-delivery of the WNT1 ligand stimulated the pathway as confirmed by a reporter assay but it also resulted in an increase in endogenous Wnt targets.

Overexpression of the WNT1 ligand in other systems results in hyperproliferation and tumor formation [26]. Consistent with these reports, we observed an expansion of the intestinal crypt proliferative zone (Figure 3e). Ki67 antibodies recognize proliferating cells in the intestine which are confined to the crypt during normal adult intestine homeostasis [17]. In lentiviral-mediated WNT1-expressing intestines, the proliferative zone was expanded beyond the crypt-villus junction (solid white line, Figure 3e). This indicated that moderate and local influence of exogenously expressed WNT1 ligand in the mesenchyme resulted in hyperproliferation. Further, 67% of the WNT1 infected mice developed at least one polyp (Figure 3f, g).

In summary, our findings show that lentiviral vectors can be used for modulation of signaling pathways that require a paracrine-expressed ligand. A single intraperitoneal injection resulted in mesenchymal expression in the intestine, as well as other gastrointestinal and blood-derived organs. Our system offers a superior approach for delivering ligand expression to the mesenchyme negating the need for technically challenging *in utero* surgeries, bone marrow transplants, or complicated mating schemes for genetic models. In addition, our approach offers the advantage of easily identifying infected cells with GFP expression driven off the bicistronic message, allowing for phenotypic changes in the epithelium to be monitored at a cellular level. The ability to track infected cells allows for comparisons of an elicited phenotype with normal adjacent regions within the local intestinal microenvironment. Recent reports using shRNA or siRNA-containing lentiviruses [27] suggest that our scheme could also be used for knock-down of ligand transcripts to extend our understanding of the factors that regulate the intestinal stem cell or other organ stem cells that are modulated by paracrine signaling. We envision that lentiviruses will become valuable tools to study Wnt and other signaling pathways, allowing us to dissect ligand-receptor interactions as we seek to further understand the intricacies of signaling within the mouse during both development and in disease.

## Competing Financial Interests

The authors declare that they have no competing interests.

## Appendix

### Lentivirus Production Protocol

#### Materials

Lentiviral plasmids pSL3, pSL4, pSL5, and pSL35

FuGene 6 (Roche)

T75 cell culture flasks

HEK 293T/17 cells

OPTI-MEM serum-free media (Invitrogen)

Dulbecco's minimal essential medium (DMEM) + 10% fetal bovine serum + Pen/Strep 100 μg/ml

0.45 μM self-contained conical tube filters

40 mL Ultra Clear Beckman centrifuge tube

Beckman SW28 swinging bucket rotor

Beckman ultracentrifuge

Dulbecco's phosphate buffered saline

#### Production

1. Start with 3× T75 flasks of 50–80% confluent HEK 293T/17 cells in 11 mL DMEM + 10% fetal bovine serum + Pen/Strep 100 μg/ml.

• Hint: Seed T75 flasks at 4.5 × 10^6^ the day before transfection. Total volume should be 11 mL per dish.

2. Make a transfection master mix:

• Combine lentiviral plasmids in a microcentrifuge tube 2 μg pSL3 (envelope), 8 μg pSL4 (packaging), 4 μg pSL5 (Rev), and 4 μg pSL35 (transducing).

• Add 1,746 μl serum-free Opti-MEM to a sterile 15-mL conical tube (Falcon). Pipet 54 μl FuGENE 6 transfection reagent directly to the serum-free medium. FuGene will bind to the plastic of the tube, so be sure not to touch the sides of the tube. Invert tube to mix.

• Add the plasmid DNA mixture to the FuGene/media mix. Gently tap the tube to mix but do not vortex. Incubate for 15 min at room temperature.

3. Add 600 μl of the transfection master mix dropwise to the flask. Swirl the flask to ensure even dispersal. Incubate at 37 C in 5% CO_2_.

4. At 48 h post-transfection, collect supernatants into a 50-mL conical tube (Falcon) from the dishes, replacing with 11 mL fresh DMEM +10% fetal bovine serum + Pen/Strep 100 μg/ml per dish. If a fluorescent marker (such as GFP) is present, transfection efficiency can be confirmed visually. Repeat at 72 h post-transfection.

5. Spin both tubes of collected media for 5 min at 2,000 rpm in a table top centrifuge to remove large cellular debris.

6. Clear the supernatant of cell debris by filtering through a 0.45-μm filter.

7. Transfer virus-containing media to two 40-mL ultraclear Beckman tubes using a swinging bucket SW28 rotor and ultracentrifuge for 90 min at 4 C, 25,000 rpm.

8. Remove tubes and decant supernatant. Invert tubes over a sterile Kimwipe to allow remaining media to drain.

• Hints: save media until virus is titered. Allow to drain approximately 5 min.

9. Add 300 μl of cold 1× Dulbecco's phosphate buffered saline to the viral pellet in each tube. Seal tubes with parafilm and place at 4°C for at least 12 h to dissolve the pellet.

10. Pipette gently to resuspend the pellet and combine the two collections in a single microcentrifuge tube. The virus can be aliquoted, titered, and stored at -80°C until use.

### Lentiviral Titer Using a Fluorescent Protein

1) Day 1: seed 8 × 10^5^ cells in a 6-well plate in 2 mL DMEM + 10% fetal bovine serum + Pen/Strep per well and incubate overnight at 37 C, 5% CO_2_.

2) Day 2: add 10 μl of tenfold serially diluted lentivirus (such that final volumes of added lentivirus are 10, 1, 0.1, 0.01, and 0.001 μl) to individual wells of the 6-well plate, leaving one well uninfected as the negative control.

3) Day 5 (3 days post-infection): check for GFP fluorescence on inverted microscope fitted with a fluorescent lamp.

4) Remove media and wash each well gently with 2 mL warm PBS. Add 1 mL cold PBS to each well. Pipette up and down ten times to lift cells and ensure single cell suspension. Collect each well into separate microcentrifuge tubes and centrifuge at 3,000 g aspirate supernatant and resuspend each sample in 1 mL of Opti-MEM.

5) Analyze cells for GFP expression by flow cytometry.

Calculate viral titer based on percentage of GFP-expressing cells: (cell count × percentage GFP-expressing cells)/μl of viral stock = viral titer (IU = infectious units)
